# *Leishmania infantum* and *Dirofilaria immitis* infections in Italy, 2009–2019: changing distribution patterns

**DOI:** 10.1186/s13071-020-04063-9

**Published:** 2020-04-15

**Authors:** Jairo Mendoza-Roldan, Giovanni Benelli, Rossella Panarese, Roberta Iatta, Tommaso Furlanello, Frederic Beugnet, Andrea Zatelli, Domenico Otranto

**Affiliations:** 1 Dipartimento di Medicina Veterinaria, Università degli Studi di Bari, Bari, Italy; 2 Department of Agriculture, Food and Environment, University of Pisa, Pisa, Italy; 3 San Marco Veterinary Clinic and Laboratory, Veggiano, Padova Italy; 4 Boehringer Ingelheim Animal Health, Lyon, France; 5 Faculty of Veterinary Sciences, Bu-Ali Sina University, Hamedan, Iran

**Keywords:** *Aedes* mosquitoes, Canine vector-borne diseases, *Dirofilaria immitis*, *Leishmania infantum*, Sandflies

## Abstract

**Background:**

For long time, canine leishmaniosis (CanL) was considered endemic in the southern, central, and insular regions of Italy, whereas heartworm disease (HW) caused by *Dirofilaria immitis* was considered endemic in the northern region and in the swampy Po Valley. Following the reports of new foci of both diseases, in this study we update the distribution patterns and occurrence of new foci of CanL and HW discussing the main drivers for the changes in the epidemiology of these two important zoonotic canine vector-borne diseases.

**Methods:**

Based on the statistical analyses of serological assays (*n* = 90,633) on *L. infantum* exposure and *D. immitis* infection performed by two reference diagnostic centres in Italy over a ten-year period (2009–2019) irrespective of the anamnesis of dogs. The distribution patterns of both parasites are herein presented along with the occurrence of new foci.

**Results:**

Results highlighted the changing distribution patterns of *L. infantum vs D. immitis* infection in Italy. CanL is endemic in some areas of northern regions and HW has endemic foci in central and southern regions and islands. Significant differences in *L. infantum* exposure and HW infection prevalence among the study macroareas were detected. The overall results of the positive tested samples were 28.2% in southern Italy and islands, 29.6% in central Italy and 21.6% in northern Italy for *L. infantum* and 2.83% in northern Italy, 7.75% in central Italy and 4.97% in southern Italy and islands for HW. HW positivity significantly varied over years (*χ*^2^ = 108.401, *df* = 10, *P* < 0.0001), gradually increasing from 0.77% in 2009 to 8.47% in 2016–2017.

**Conclusions:**

New potential epidemiological scenarios are discussed according to a range of factors (e.g. environmental modifications, occurrence of competent insect vectors, transportation of infected animals to non-endemic areas, chemoprophylaxis or vector preventative measures), which may affect the current distribution. Overall, the results advocate for epidemiological surveillance programmes, more focussed preventative and control measures even in areas where few or no cases of both diseases have been diagnosed.
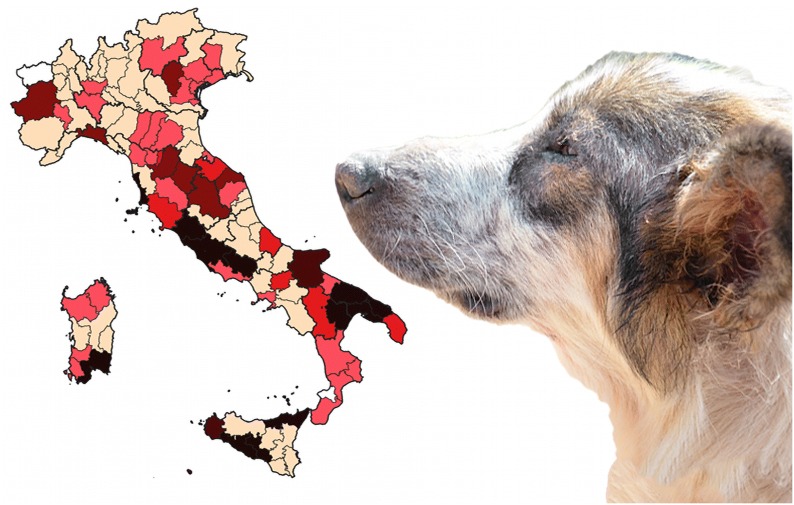

## Background

In the last decades, canine vector-borne diseases (CVBDs) have been expanding worldwide due to several factors linked with increase in pet travelling along with owners, relocation of sheltered animals from endemic to previously non-endemic regions, as well as to the modification of the ecology of arthropod vectors and, importantly, environmental modifications [1–3]. Therefore, the spreading of new parasites and pathogens, and related infections, in previously non-endemic geographical areas poses major concerns to veterinary practitioners and, in the case of zoonotic ones, to public health officials [2]. The protozoan *Leishmania infantum* Nicolle and the nematode *Dirofilaria immitis* Leidy represent paradigmatic examples of the modification in the distribution of the diseases they cause (i.e. canine leishmaniosis, CanL, and heartworm disease, HW). The expansion of the above diseases has been related to the distribution of their vectors (i.e. for CanL, sandflies of the genus *Phlebotomus* in the Mediterranean region; for HW, several mosquito species, belonging to the genera *Aedes*, *Anopheles* and *Culex* [4–7]).

In specific geographical contexts, such as Italy, where both CanL and HW have been endemic for long time [8], their ecology and distribution have been studied and new foci were reported by a retrospective analysis focusing on the period from 1990 to 2009 [1]. Indeed, until 1990, CanL was considered endemic in the southern, central and insular regions of Italy, whereas HW was considered endemic in the northern region and in the Po Valley [9, 10]. Sporadic case reports suggested that the distribution of both CVBDs has been changing, in that, dirofilariosis expanded towards the southern regions and CanL to the northern regions [1]. The spreading of dirofilariosis in previously non-endemic areas has been facilitated by the absence of chemoprophylaxis measures in the canine population, by using macrocyclic lactones (ML), as routinely performed in endemic areas of northern Italy [11]. Indeed, the only species of filarioid historically diagnosed in southern Italy have been *Acanthocheilonema reconditum* and *Dirofilaria repens*, causing less pathogenic subcutaneous filariosis [12, 13].

Meanwhile, the perception of clinicians and parasitologists has most likely changed, resulting into an increase of the requests for diagnostic tests of *D. immitis* infection in central-southern Italy, as well of *L. infantum* exposure in northern regions [8]. In this scenario, the aim of the present study is to analyse the results of serological assays performed by two reference diagnostic centres in Italy over a ten-year period (2009–2019), therefore providing a picture of the possible distribution patterns of CanL and HW.

## Methods

### Databases of serological tests in dogs from Italy

**Fig. 1:**
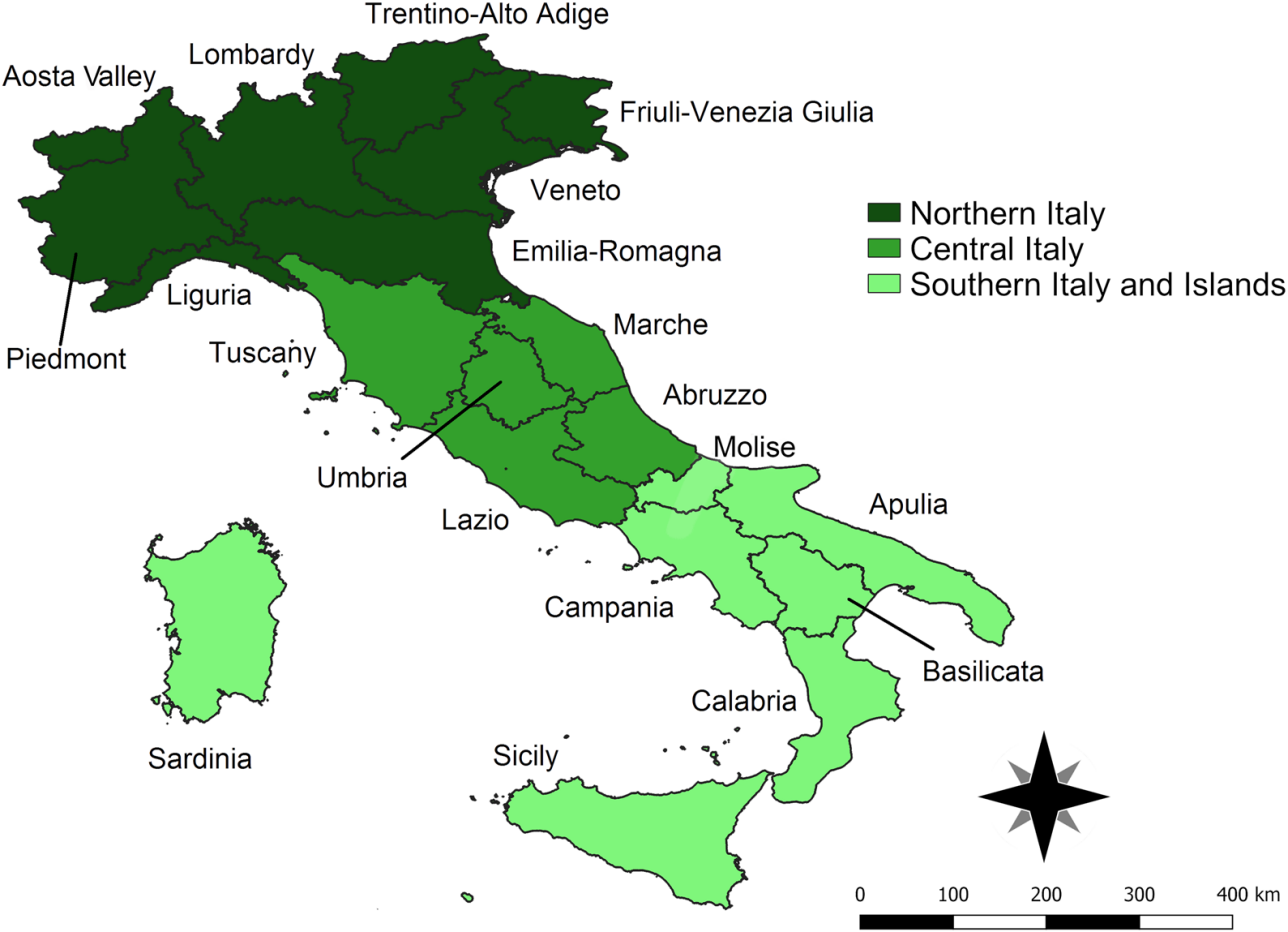
Map of Italy showing the three main areas with their respective administrative regions

Databases from two diagnostic reference centres (hereafter reported as database A and B) were analysed (total number of serum samples = 90,633). Italian macroareas were defined, according to the geopolitical classification, as northern, central and southern/islands macroareas (Fig. 1).

The compiled data were included in the same database to observe significant variations on positive serological results. The database A included 64,375 records of dogs collected in a 10-year period (2009–2019) throughout the Italian Peninsula, including information regarding breed, sex and age (data not shown). Different serological (Novatec® kit for CanL and Dirocheck Zoetis® for HW) enzyme-linked immune sorbent assay (ELISA) tests were used. Out of 64,375 records, 78 were excluded due to uncertainty of the origin of the samples.

The second dataset (B) included information of 26,258 of dogs examined from northern Italy (i.e. Emilia Romagna, Lombardia, Trentino-Alto Adige and Piedmont), central Italy (i.e. Lazio and Tuscany), and mainly from southern Italy and islands (i.e. Basilicata, Calabria, Campania, Apulia, Sicily and Sardinia) in a five-year period (2015–2019). This dataset also comprised information regarding breed, sex and age (data not shown), and serological (VetLine® *Leishmania* for CanL, sensitivity and specificity of > 98%; and Filarcheck 96® for HW, sensitivity of 97.6%, specificity of 100%) ELISA, and an indirect semi-quantitative immunofluorescence test (MegaFLUO LEISH® for leishmaniosis, sensitivity of 96.9%, specificity of 98.7%) with positive or negative results. Out of 26,258 records, 70 were excluded due to uncertainty of the origin of the samples, and other 423 samples from the northern and central regions were not considered for the statistical analysis given that the data were biased by transportation of positive animals from endemic areas. However, for all included samples in both datasets no anamnestic data were available.

### Statistical analyses

Differences in the prevalence of *L. infantum* exposure and *D. immitis* infection in the samples available from northern, central and southern Italy over time were analysed by JMP 9 (SAS) by using weighted generalized linear models (GLZ) with a binomial distribution to test model positive and negative serological outcomes. For each parasite, a GLZ with two fixed factors was used to assess significant differences in *L. infantum* or *D. immitis* positivity (i.e. prevalence) among the study macroareas over years: *y* = *Xß* + *ε*, where *y* is the vector of the observation (i.e. serological outcome: positive = 1, negative = 0), *X* is the incidence matrix, *ß* is the vector of fixed effects (the study macroarea: northern, central or southern Italy and islands; and years), and *ε* is the vector of the random residual effects (*P* = 0.05).

Then, a dataset was created for each study macroarea and a GLZ with two fixed factors was used to evaluate significant differences in *L. infantum* or *D. immitis* positivity between the regions and years within a given macroarea; the structure of the GLZ was identical to the above described one, with two fixed effects (i.e. the study region and year). A *P*-value of 0.05 was used as a threshold to assess significant differences among values. To verify that the changing distribution patterns of *L. infantum* and *D. immitis* were not random, a contingency analysis assessing the relationship between the *L. infantum* and *D. immitis* positivity in the various macroareas, regions and study years was also carried out [14].

Serological data were presented in terms of annual and cumulative prevalence; distribution maps of cumulative positive cases for *L. infantum* and *D. immitis* were generated using QGIS version 3.4.4-Madeira [15].

## Results

Overall, the number of serological tests performed for *L. infantum* (*n* = 80,309) in the three areas of Italy is reported in Table 1, being higher in southern Italy (37.7% of all tests performed) than in central Italy (31%), and northern Italy (24.7%). Conversely, the overall number of tests requested for the diagnosis of *D. immitis* throughout Italy (*n* = 10,324) was significantly lower (11.3% of all the requested serological tests) than that for *L. infantum*, with a relative high number in northern Italy (51.7% of all tests performed) and the lowest in southern Italy (Table 1).

**Table 1:** Number (*n*) and percentage of serological tests positive for canine leishmaniosis and hearthworm disease in three main macroareas of Italy

Species	North *n* (%)	Centre *n* (%)	South and islands *n* (%)
*Leishmania infantum*	4664 (21.6) (Total: 21,545)	7801 (29.6) (Total: 26,128)	9208 (28.2) (Total: 32,610)
*Dirofilaria immitis*	151 (2.8) (Total: 5335)	243 (7.5) (Total: 3119)	93 (4.9) (Total: 1866)

**Fig. 2:**
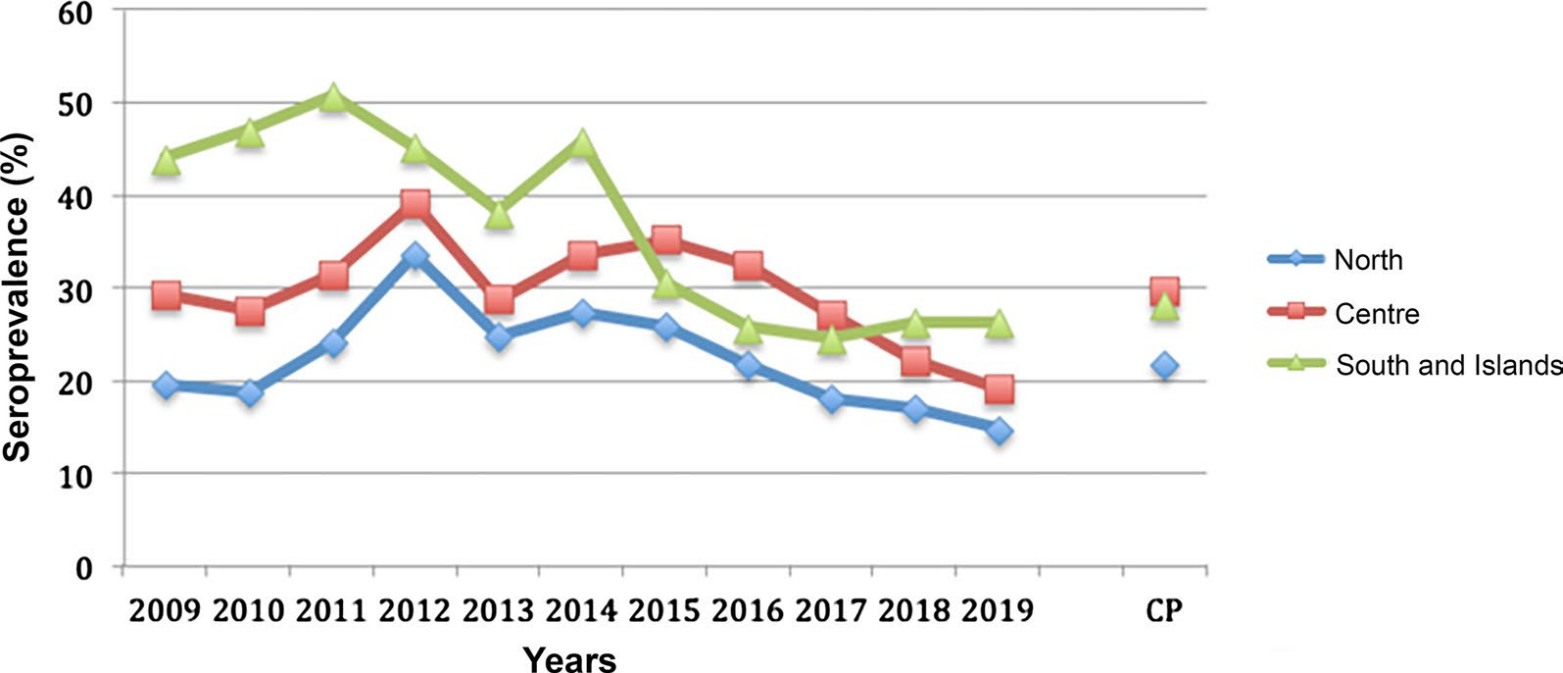
Pattern of mean annual seroprevalence of canine leishmaniosis in the three main macroareas of Italy. The cumulative prevalence (CP) is also shown

**Fig. 3:**
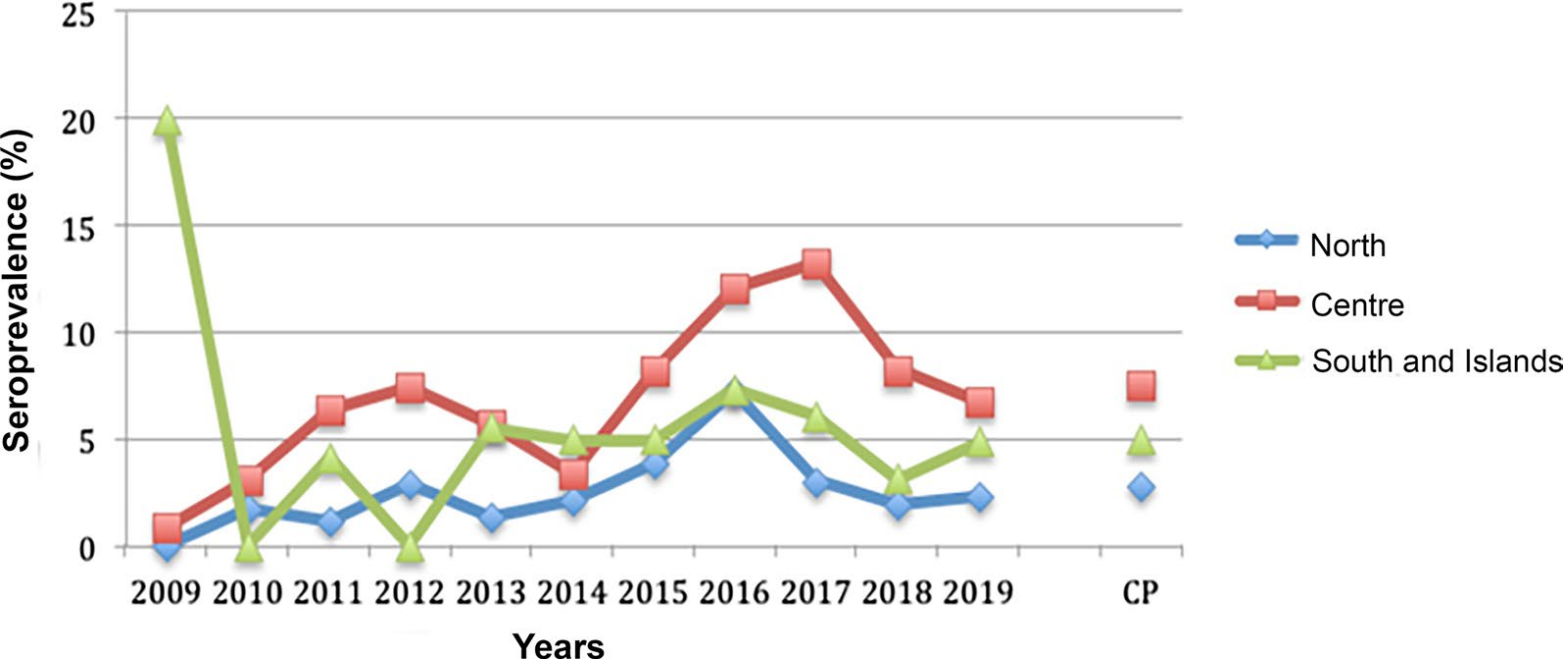
Pattern of mean annual seroprevalence for *Dirofilaria immiti*s in the three main macroareas of Italy. The cumulative prevalence (CP) is also shown

Data showed a significant difference in *L. infantum* prevalence between the different study macroareas (*χ*^2^ = 218.564, *df* = 2, *P* < 0.0001), with an overall positivity of 28.2% in southern Italy and islands, 29.68% in central Italy and 21.62% in northern Italy (Table 1, Fig. 2). The impact of the study year on *L. infantum* results in Italy was also significant (*χ*^2^ = 559.846, *df* = 10, *P* < 0.0001), with values > 30% in 2011, 2012, 2014 and 2015. *Dirofilaria immitis* prevalence showed significant differences between the study macroareas (*χ*^2^ = 114.879, *df* = 2, *P* < 0.0001), being of 2.83% in northern Italy, 7.75% in central Italy and 4.97% in southern Italy and islands. On the whole Italian territory, the number of HW positive tests significantly varied over years (*χ*^2^ = 108.401, *df* = 10, *P* < 0.0001), gradually increasing from 0.77% in 2009 to values ranging between 5.19–8.47% in 2016–2017 (Table 1, Fig. 3).

A detailed analysis of the trends of prevalence for *D. immitis* and *L. infantum* over time in the three Italian macroareas was provided by assessing the impact of the study year and region on prevalence for both pathogens. In northern Italy, *L. infantum* prevalence showed significant differences over the study years (*χ*^2^ = 286.277, *df* = 10, *P* < 0.0001), being highest in 2012 and 2014 (i.e. 33.51% and 27.27%, respectively) (Fig. 2). Notably, significant variations in tests positive for *L. infantum* among the various northern Italy regions were recorded (*χ*^2^ = 190.657, *df* = 7, *P* < 0.0001), with the highest values in Piedmont, Trentino Alto Adige, Aosta Valley and Friuli Venezia Giulia (28.93%, 27.59%, 27.40% and 27.17%, respectively). The study year and region significantly impacted the results of *D. immitis* in northern Italy (*χ*^2^ = 56.954, *df* = 10, *P* < 0.0001; and *χ*^2^ = 40.555, *df* = 7, *P* < 0.0001, respectively), with the highest prevalence observed in 2016 (7.20%) (Fig. 3). The highest prevalence rate of *D. immitis* were recorded in Aosta Valley(11.36%), Trentino Alto Adige (7.41%) and Piedmont (6.29%), while the lowest was found in Veneto (2.12%).

In central Italy, both the study year and region led to significant differences in *L. infantum* prevalence (*χ*^2^ = 371.252, *df* = 10, *P* < 0.0001; and *χ*^2^ = 609.769, *df* = 5, *P* < 0.0001, respectively). It was more than 30% in 2012, 2014, 2015 and 2016 (Fig. 2), with the highest values in Lazio (38.52%), followed by Umbria (35.61%) and Abruzzo (34.10%). Positive results for *D. immitis* were also affected by the study year (*χ*^2^ = 55.333, *df* = 10, *P* < 0.0001), showing values of > 10% in 2016 (i.e. 12.1%) and 2017 (i.e. 13.32%) (Fig. 3). Significant differences in the prevalence for *D. immitis* were noted in central Italy (*χ*^2^ = 80.975, *df* = 4, *P* < 0.0001, respectively) with the highest values recorded in Tuscany (11.48%) and Marche (7.84%).

In southern Italy and islands, *L. infantum* positive tests were significantly different among the study years (*χ*^2^ = 201.963, *df* = 10, *P* < 0.0001), with values ranging from 24.55% (2017) to 50.66% (2011) (Fig. 2). *Leishmania infantum* results in this macroarea also showed significant differences between regions (*χ*^2^ = 642.949, *df* = 6, *P* < 0.0001) with the highest values recorded in Molise (54.26%), Sicily (50.18%) and Sardinia (38.34%). On the other hand, the study region did not play a significant role impacting *D. immitis* prevalence, even if a trend was observed (*χ*^2^ = 10.723, *df* = 6, *P* = 0.10, respectively). Indeed, the largest number of analysed samples was from Apulia (*n* = 1608), followed by Basilicata (*n* = 80) and Sardinia (*n* = 70). Considering regions with sample size > 40, the largest number of positive tests were from Sardinia and Apulia (10% and 4.73%, respectively). The effect of the study year was not significant (*χ*^2^ = 16.145, *df* = 10, *P* = 0.136) due to the limited number of samples examined during 2009–2014 (i.e. *n* = 117), at variance with the larger number of samples during 2015–2019 (i.e. *n* = 1729).

**Fig. 4:**
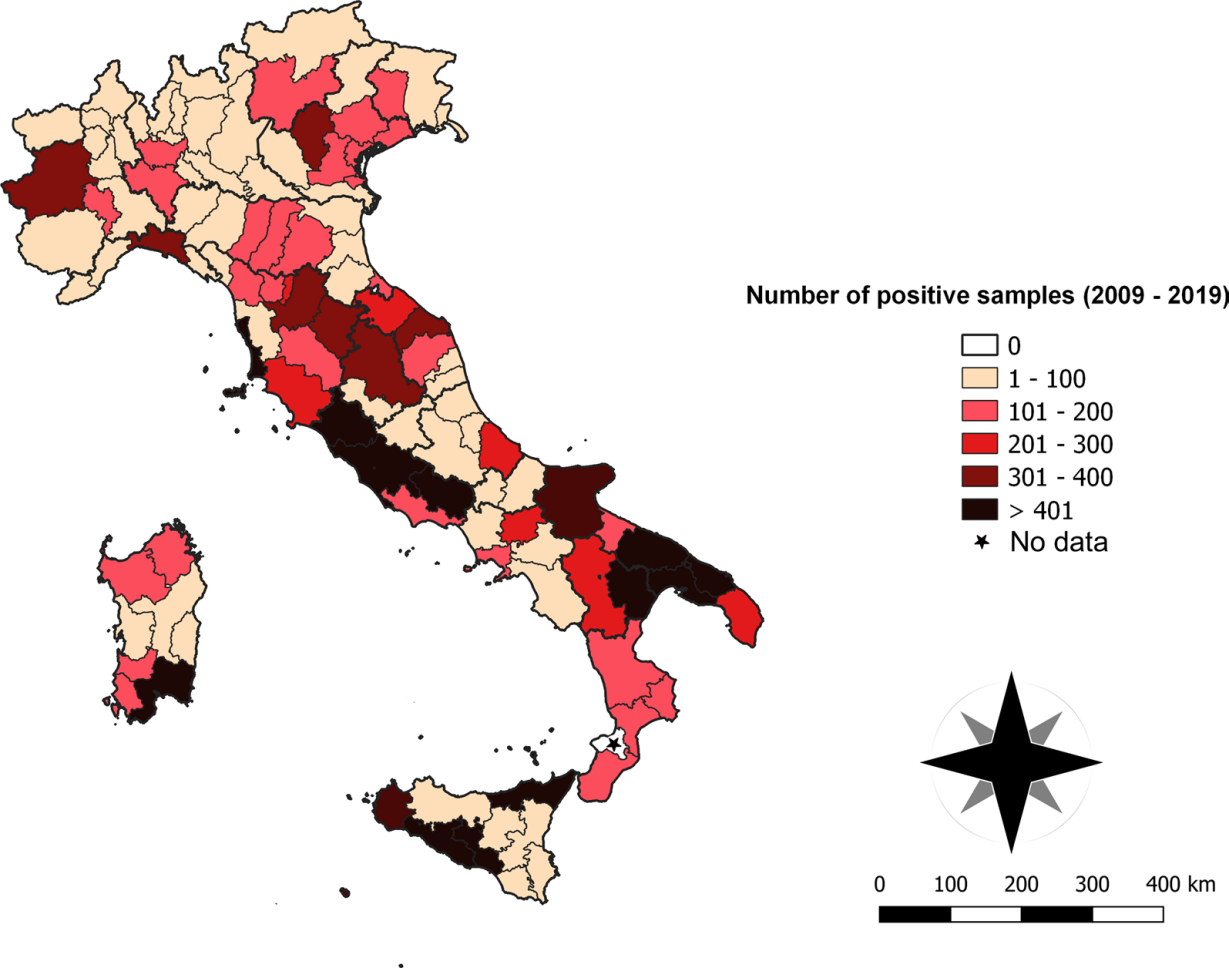
Distribution map with number of cases (2009–2019) per province of CanL in Italy

Contingency results obtained analysing the separate datasets for each study macroarea are given in Additional file 1: Table S1. Overall, the observed changing distribution patterns of *L. infantum* and *D. immitis* in Italy were not random or due to a biased sampling over the different areas and years (*L. infantum*, macroarea: *χ*^2^ = 486.62, *df* = 2, *P* < 0.0001; year: *χ*^2^ = 827.903, *df* = 10, *P* < 0.0001, respectively; *D. immitis*, macroarea: *χ*^2^ = 104.545, *df* = 2, *P* < 0.0001; year: *χ*^2^ = 99.070, *df* = 10, *P* < 0.0001, respectively). Serological *L. infantum* results had a similar cumulative prevalence throughout the Italian territory (Fig. 2), showing an annual slight decrease in all the regions, from 2015 to 2018. *Leishmania infantum* was widely distributed throughout the Italian Peninsula, with a number of positive animals > 400 in the central (Lazio and Tuscany) and southern (Apulia and Basilicata) regions as well as in both islands (Sardinia and Sicily). In addition, provinces with > 300 positive samples were in the northern regions (i.e. Turin, Piedmont and Vicenza, Veneto) (Table 1, Fig. 4).

**Fig. 5:**
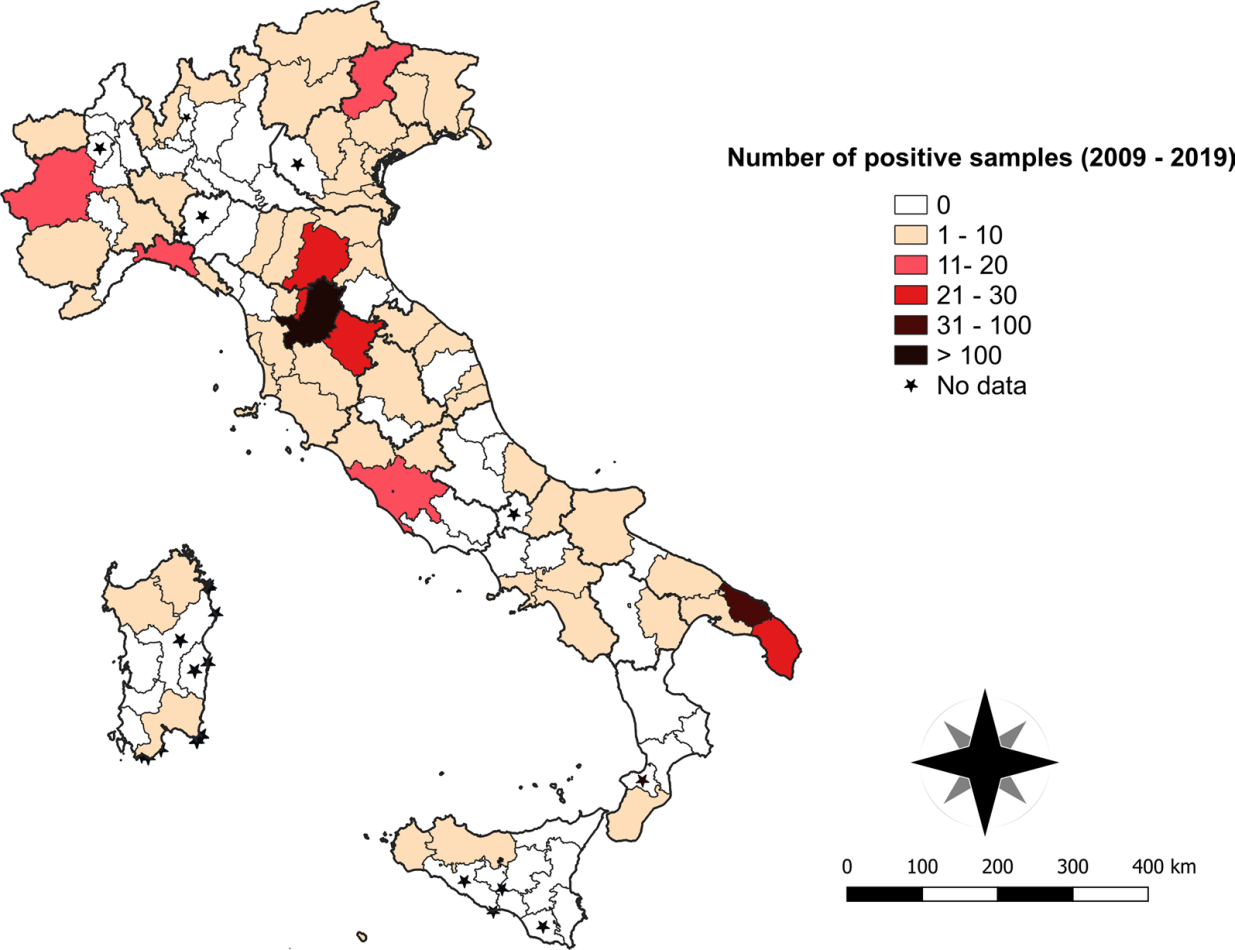
Distribution map with number of cases (2009–2019) per province of HW in Italy

The highest percentage of positive tests of *D. immitis* was registered in the central regions (i.e. Tuscany and Lazio) followed by the southern and northern regions with annual variation patterns throughout the observation period (Fig. 3). An overall lower number of samples was positive for *D. immitis* compared to *L. infantum*, with > 10 positive cases recorded in three provinces in the northern Italy (i.e. Turin, Piedmont; Genova, Liguria; and Belluno, Veneto). In central Italy, the province with the greatest number of HW cases (i.e. Florence, Tuscany) was surrounded by provinces with more than 20 positive samples (i.e. Bologna, Emilia Romagna; Arezzo and Prato, Tuscany). The largest number of positive samples in southern Italy was recorded in the Apulia region (i.e. Brindisi and Lecce provinces). No data were available for four provinces of the North (i.e. Biella, Piedmont; Lecco, Lombardy; Piacenza, Emilia Romagna; and Verona, Veneto), for two provinces of southern Italy (i.e. Isernia, Molise; Vibo-Valentia, Calabria), and many provinces of the islands (i.e. Nuoro and Oristano, Sardinia; Agrigento, Caltanissetta and Ragusa, Sicily) (Table 1, Fig. 5).

## Discussion

Based on the results of this large dataset (*n* = 90,633) of serological assays presented herein, the positive tests for *L. infantum* and *D. immitis* varied over the last 10 years’ period (2009–2019), also in comparison with data available from the prior decade (1999–2009) [1]. Indeed, the number of positive serum samples of *L. infantum* in northern Italy increased in the examined 10 years’ period (2009–2019) from 2.1% (1999–2009; [1, 16]) to 21.6%. The results also indicate that the number of cases positive for *L. infantum* has increased progressively in the past decade in the northern Italian regions, with an overall prevalence higher than that recorded in previous studies [1, 17]. For example, in the province of Bologna (Emilia Romagna) the seroprevalence increased from 6.6% (i.e. 16 out of 245 animals examined in the period 2007–2009 [17]) up to 17.16% (i.e. 217 out of 1264 tests in this study). Overall, *L. infantum* exposure has spread progressively in the past decades from the endemic southern regions towards northern regions, making the whole Italian Peninsula endemic for this infection. Under these circumstances, the effect of relocation of infected animals from the South to the North of Italy could not be fully assessed in the study period. Nonetheleess, the northward spread of the main sand fly vectors, i.e. *Phlebotomus perniciosus* (Newstead) and *Phlebotomus neglectus* Tonnoir, which are now established in several provinces of the northern macroarea [16, 18, 19], supports the evidence of *L. infantum* endemicity and the occurrence of new foci. In addition, clinicians’ perception and awareness of the presence of CanL in non-endemic areas seem to be increased in the northern regions, with more than 21,545 tests performed, whereas the South remains the area with the largest number of tests requested (Table 1).

On the other hand, the cumulative prevalence of *D. immitis* infection greatly increased in central (7.7%) and southern Italy and islands (5%), being higher than in the North (2.8%), which was historically considered the sole endemic area [1, 20]. The decreased prevalence of *D. immitis* infection in northern Italy regions could be a consequence of clinicians’ awareness of the disease and thus of the continuous usage of chemoprophylactic programmes in this area. Nonetheless, resistance to ML has been demonstrated in *D. immitis* populations from this macroarea [21], which may represent a potential issue (not yet proven) for emergence of resistant strains. Moreover, the low number of tests performed for *D. immitis* infection (10.5% of all the requested serological tests) throughout the Italian Peninsula also indicates the scant awareness of the occurrence of the disease, especially in the southern regions (Table 1). Given the increased prevalence in the Centre, the South and islands, clinicians should consider the occurrence of HW cases in non-endemic areas.

Furthermore, our analyses showed that the distribution patterns of *L. infantum* and *D. immitis* in Italy are related to a significant relationship between *L. infantum* /*D. immitis* positive results of the tests, the geographical provenience (i.e. macroarea) and study years (2009–2019). Conversely, a major hindrance of this study could be that the anamnesis of animals was not available (e.g. their travelling history from/to historically endemic areas for both diseases) [5, 22].

However, the clustering of positive samples in some spots of *L. infantum* exposure [e.g. the provinces of Turin (Piedmont) and Vicenza (Veneto), northern Italy; Fig. 3] and of *D. immitis* infection [e.g. in Brindisi and Lecce (Apulia), southern Italy; Fig 4] is consistent with previous reports. Endemic foci of CanL in the same provinces of Veneto [23] and Piedmont [24] confirmed the above mentioned epidemiological picture with a competent vector of *L. infantum* (i.e. *P. perniciosus*) captured in entomological surveys performed in that area [19]. In addition, other studies reported cases of CanL and human visceral leishmaniasis diagnosed in the same area, along with their vectors, *P. perniciosus* and *P. neglectus* [25].

The southward changing pattern of HW has been detailed in previous studies [26–29]. Hyperendemic *D. immitis* infection has been recorded in sheltered dogs from Apulia region (southern Italy) with the highest prevalence of infection (i.e. 44.2%) in Europe [30]. The low prevalence of HW in Sicily and Sardinia could be explained by the small number of tests requested or no tests performed in many provinces from these islands. Indeed, earlier studies illustrated the islands to have a large number of diagnosed HW [31] as they are suitable for *Dirofilaria* spp. to thrive [13]. Specifically, Sardinia has the environmental, climatic and human activities (e.g. tourism with animal transportation) that could allow these nematodes to spread [5, 30]. Finally, central Italy showed a distribution pattern on which both pathogens are highly prevalent. In this macroarea, the prevalence of *L. infantum* was higher (29.6%) of that indicated in previous surveys (up to 10%) [32, 33]. Hence, central Italian regions are an important pathway for the spreading of CanL from southern to northern.

## Conclusions

The large number of data analized strongly supports the above figure of distribution of *L. infantum* and *D. immitis*. Overall, results highlighted the changing distribution patterns of CanL *vs* HW in Italy over a 10-year period (2009–2019) in which the number of cases of CanL has increased in the northern Italy regions, and HW has endemic foci in the central and southern Italy and in islands. Moreover, given the number of tests requested, the overall clinicians’ awareness in northern Italy is increasing for CanL and diminishing for HW. Data presented suggest that veterinarians and public health officials should be aware that CanL and HW are distributed throughout the country, thus epidemiological surveillance, preventative and control measures should be carried out to protect dogs from those CVBDs and reduce the risk of infection in humans.

## Supplementary information

**Additional file 1: Table S1.** Contingency analysis summarizing the relationships between leishmaniosis (CanL) and dirofilariosis (HW) prevalence and the study year and region; the asterisk indicates a significant relationship with a study factor (*P* < 0.05); n.s., not significant (*P* > 0.05).

## Data Availability

All data generated or analyzed during this study are included in this published article and its additional file.

## Abbreviations

CVBDs: canine vector-borne diseases
CanL: canine leishmaniosis
HW: heartworm disease
CI: confidence interval
