# Supplementary material for: Leishmania infantum and Dirofilaria immitis infections in Italy, 2009–2019: changing distribution patterns
Source: Parasit Vectors. 2020 Apr 15;13:193. doi: 10.1186/s13071-020-04063-9 (PMC7161282; doi:10.1186/s13071-020-04063-9)
Supplement: Supplementary file 1 — Additional file 1: Table S1. Contingency analysis summarizing the relationships between leishmaniosis (CanL) and dirofilariosis (HW) prevalence and the study year and region; the asterisk indicates a significant relationship with a study factor (P < 0.05); n.s., not significant (P > 0.05). [file 13071_2020_4063_MOESM1_ESM.docx]

**Additional file 1: Table S1.** Contingency analysis summarizing the relationships between leishmaniosis (CanL) and dirofilariosis (HW) prevalence and the study year and region; the asterisk (“*”) indicate a significant relationship with a study factor, n.s.=not significant (*p*>0.05).

| **CVBD** | **Macroarea** | **Tested factor** | | | | | |
| --- | --- | --- | --- | --- | --- | --- | --- |
|  |  | **Year** | | | **Region** | | |
|  |  | *χ^2^* | *d.f.* | *p* | *χ^2^* | *d.f.* | *p* |
| CanL | northern Italy | 327.551 | 10 | <0.0001* | 231.932 | 7 | <0.0001* |
|  | central Italy | 334.187 | 10 | <0.0001* | 572.704 | 4 | <0.0001* |
|  | southern Italy and Islands | 500.113 | 10 | <0.0001* | 990.198 | 6 | <0.0001* |
| HW | northern Italy | 56.054 | 10 | <0.0001* | 39.655 | 7 | <0.0001* |
|  | central Italy | 73.839 | 10 | <0.0001* | 99.481 | 4 | <0.0001* |
|  | couthern Italy and Islands | 12.568 | 10 | 0.323 n.s. | 7.145 | 6 | 0.307 n.s. |
